# Transient dynamic mechanical properties of resilin-based elastomeric hydrogels

**DOI:** 10.3389/fchem.2014.00021

**Published:** 2014-04-28

**Authors:** Linqing Li, Kristi L. Kiick

**Affiliations:** ^1^Department of Materials Science and Engineering, University of DelawareNewark, DE, USA; ^2^Biomedical Engineering, University of DelawareNewark, DE, USA; ^3^Delaware Biotechnology InstituteNewark, DE, USA

**Keywords:** resilin-like polypeptide, elastomer, hydrogel, biomaterials, vocal fold

## Abstract

The outstanding high-frequency properties of emerging resilin-like polypeptides (RLPs) have motivated their development for vocal fold tissue regeneration and other applications. Recombinant RLP hydrogels show efficient gelation, tunable mechanical properties, and display excellent extensibility, but little has been reported about their transient mechanical properties. In this manuscript, we describe the transient mechanical behavior of new RLP hydrogels investigated *via* both sinusoidal oscillatory shear deformation and uniaxial tensile testing. Oscillatory stress relaxation and creep experiments confirm that RLP-based hydrogels display significantly reduced stress relaxation and improved strain recovery compared to PEG-based control hydrogels. Uniaxial tensile testing confirms the negligible hysteresis, reversible elasticity and superior resilience (up to 98%) of hydrated RLP hydrogels, with Young's modulus values that compare favorably with those previously reported for resilin and that mimic the tensile properties of the vocal fold ligament at low strain (<15%). These studies expand our understanding of the properties of these RLP materials under a variety of conditions, and confirm the unique applicability, for mechanically demanding tissue engineering applications, of a range of RLP hydrogels.

## Introduction

Elastomeric biomaterials have long been targets for application in the engineering of mechanically active tissues, and recently introduced resilin-like polypeptides (RLPs) may have unique suitability for such applications. In particular, our laboratories have been developing RLPs for application in the treatment of vocal fold pathologies. Human phonation occurs in the larynx by airflow-induced, self-sustained oscillation of a layered connective tissue called the vocal fold lamina propria (LP), which is the major vibratory tissue activated during phonation (Titze, 1984, 1989; Jiang and Tao, 2007). The vibration of the vocal folds is controlled actively by an aerodynamic driving force (e.g., lung pressure), vocal fold positioning, and non-linear interactions, but most importantly depends on the viscoelasticity of the connective tissue of the vocal fold LP; (Riede et al., 2011) these mechanical properties are important both in terms of short transient responses and longer time-dependent behavior (Titze, 1988; Chan and Titze, 1999, 2000; Chan, 2004; Klemuk and Titze, 2004). Two distinct types of forces are involved in human phonation: oscillatory shear force generated from the vibration of the vocal mucosa and longitudinal stretching force derived from elongation of the vocal ligament at high pitch or large tissue deformation (Titze, 1988; Min et al., 1995; Chan and Titze, 1999, 2000). The viscoelastic properties of the vocal mucosa under shear deformation contribute to key parameters of phonation, such as the fundamental frequency, the amplitude of oscillation, and phonation threshold pressure. Titze and co-workers showed that phonation threshold pressure is directly related to the viscoelasticity of vocal fold mucosa under small amplitude oscillation (Titze, 1988). Min et al. reported mechanical properties of the human vocal ligament under longitudinal force, including the Young's modulus (33 kPa at low-strain and 600 kPa at high-strain), non-linear tensile elasticity, and hysteresis along the direction of tissue fibers (Min et al., 1995).

Mechanical stress from excessive phonation, deleterious environmental factors, and pathological conditions can disrupt the natural pliability of the vocal folds, resulting in a wide spectrum of vocal disorders and causing significant implications for individual health, social productivity, and occupational function (Zeitels et al., 2002). Surgical approaches for vocal fold augmentation or mucosal reconstruction have employed a variety of either injectable or implantable synthetic and biological materials (Hallén et al., 2001; Hirano et al., 2008; Jahan-Parwar et al., 2008; Kwon and Lee, 2008; Kishirnoto et al., 2009; Kutty and Webb, 2009b). Although improvements in voice production have been reported, limitations such as implant migration, foreign body reaction, stiffness, immunological consequences, and the need of multi-stage procedures largely have prevented restoration of functional vocal fold tissue. Recent studies aimed at the regeneration of vocal fold LP highlighted the importance of mechanical stimulation, which is directly related to the minimum subglottic air pressure needed to initiate vocal fold oscillation, and regulation of the subsequent matrix composition (Jiao et al., 2009; Kutty and Webb, 2009a; Teller et al., 2012).

Knowledge of the mechanical properties of materials targeted for vocal fold therapies is important clinically in terms of the surgical management of vocal fold disorders. However, the introduction of injectable or implantable biomaterials into the vocal fold can change the mechanical properties of the vocal fold and thus alter the mechanics of vocal fold oscillation. This is an issue of considerable clinical importance, as pathologic changes in the biomedical properties of vocal fold ECM could severely impair normal vocal fold oscillation and potentially cause severe phonation difficulties. This is particularly true in small amplitude oscillation like phonation onset and offset (Chan and Titze, 1999, 2000; Chan, 2001); these oscillations involve a surface mucosal shear wave, and frequent vibrational and shear forces are thus applied to vocal fold connective tissue. The mechanical properties of implantable/injectable biomaterials under shear are therefore particularly significant when intended for these applications.

We and others have previously reported the outstanding mechanical properties of emerging RLPs for the production of new elastomeric biomaterials, and we have been interested in coupling the biological activity of a matrix with useful high-frequency mechanical behavior for vocal fold tissue regeneration (Elvin et al., 2005; Charati et al., 2009; Li et al., 2010, 2011; Li and Kiick, 2013). The modular, recombinantly synthesized RLPs can be rapidly cross-linked via a Mannich-type condensation reaction to yield hydrogels with mechanical properties—storage shear moduli (500 Pa to 10 kPa), Young's moduli (15–35 kPa), resilience values (>90%), and storage shear moduli at high frequency (1000–2000 Pa)—that are consistent with the reported mechanical properties of native vocal fold tissues (Jiao et al., 2009; Teller et al., 2012). These RLPs also exhibit enzymatic-triggered degradation, facilitate the 2D adhesion and spreading of various cell types, and support the 3D encapsulation and survival of hMSCs *in vitro*, offering opportunities for fabricating either implantable or injectable scaffolds for vocal fold tissue therapies (Charati et al., 2009; Li et al., 2011, 2013; McGann et al., 2013).

To date, efforts have been conducted on matching the shear modulus and Young's modulus of various materials with those of natural vocal fold tissues (Thibeault et al., 2009, 2011); however, transient mechanical responses have not commonly been characterized for the materials that are proposed for vocal fold tissue engineering, particularly via oscillatory rheology methods. The investigation of transient biomechanical responses is highly relevant, however, as Zhang et al. demonstrated that the transient mechanical response of the vocal fold tissue can influence phonation processes. (Zhang et al., 2009) Given this lack of information on the dynamic transient mechanical analysis of materials proposed for vocal fold therapies, this manuscript describes both sinusoidal oscillatory shear deformation and uniaxial tensile stress-strain mechanical characterization of RLP-based hydrogels, and in particular the transient and time-dependent behavior that is of relevance for the vocal fold mucosa and vocal ligament tissues during phonation (Min et al., 1995; Zhang et al., 2007, 2009). Stress relaxation and creep experiments via oscillatory shear rheology were characterized to investigate the reduction of shear stress at a constant strain and the response of strain at a constant stress; relaxation rates were calculated from the stress relaxation data (Lv et al., 2010; Chen et al., 2012). The behavior of the RLP-based hydrogels was experimentally compared to that of polymeric PEG hydrogels. Uniaxial tensile testing of the RLP hydrogels, including stress relaxation and cyclic stress-strain deformation up to 10 cycles, was explored to determine Young's modulus, investigate hysteresis, and calculate resilience.

## Materials and methods

### Materials

Chemically competent cells of *E. coli* strain M15[pREP4] (for transformation of recombinant plasmids) and Ni-NTA agarose resin (for protein purification) were purchased from Qiagen (Valencia, CA). The tri-functional cross-linker tris(hydroxymethyl phosphine) (THP) was purchased from Strem Chemicals (Newburyport, MA). 20 kDa, amine-terminated, 4-arm PEG was purchased from Creative PEG Works (Winston Salem, NC). All other chemicals were obtained from Sigma-Aldrich (St. Louis, MO) or Fisher Scientific (Waltham, MA) and were used as received unless otherwise noted. Water was deionized and filtered through a NANOpure Diamond water purification system (Dubuque, IO).

### Expression and purification of RLP

Genes encoding the RLP polypeptide(s) were produced as described in our previous reports (Li et al., 2013); RLP protein expression and purification was also conducted as previously described (Charati et al., 2009; Li et al., 2011, 2013). The purity and molecular weight of the protein were confirmed *via* high performance liquid chromatography (HPLC), sodium dodecyl sulfate polyacrylamide gel electrophoresis (SDS-PAGE), and matrix-assisted laser desorption/ionization-time of flight mass spectrometry (MALDI-TOF-MS); the composition of the RLPs were probed via amino acid analysis. Approximately 20–30 mg of polypeptide per liter of cell culture was obtained after dialysis and lyophilization.

### RLP and PEG hydrogel formation and oscillatory rheology

The formation of RLP-based hydrogels was monitored on a stress-controlled rheometer (ARG2, TA Instruments, New Castle, DE), with a 20 mm diameter cone-on-plate geometry and a 1° cone angle with a 25 mm gap distance at 37°C. Various amounts (2, 4, and 8 mg) of RLP were dissolved in pH 7.4 PBS to attain final concentrations of 50, 100, and 200 mg mL^−1^ respectively. Stock solutions of both the RLP and the cross-linker THP (100 mg mL^−1^) were chilled on ice before mixing in order to slow the rate of the cross-linking reaction, preventing cross-linking during handling. 0.7, 1.3, and 2.6 μ L THP stock solution was added to 39.3, 38.7, and 37.4 μ L of RLP stock solutions, respectively, to yield a final solution volume of 40 μ L and 5, 10, and 20 wt% hydrogels with a 1:1 cross-linking ratio (molar ratio of lysine residues to reactive hydroxymethylphosphine (HMP) groups). For control samples, 4 mg 20 kDa, 4-arm, amine-functionalized PEG was dissolved in pH 7.4 PBS followed by the addition of 0.5 μL of THP stock solution (1:1 cross-linking ratio) to achieve the final 40 μL 10 wt% PEG hydrogel. To ensure homogeneous mixing, the mixture was vortexed gently for less than 5 s after the addition of the THP, and then followed by careful pipetting onto the rheometer Peltier plate for *in situ* rheological characterization. Time sweep studies were performed at a constant frequency (6 rad s^−1^) while frequency sweeps were conducted from 0.1 to 100 rad s^−1^ at a fixed strain amplitude of 1%. Strain sweeps were collected from 0.1 to 1000% strain at a constant frequency of 6 rad s^−1^ to determine the strain-to-break value in shear mode. Light mineral oil was applied to the perimeter of the sample to prevent evaporation of buffer over the course of the experiment. Experiments were repeated with four samples and representative data are presented.

### Oscillatory stress relaxation and creep experiments

After the hydrogels were formed as described above, stress relaxation and creep analysis were performed on RLP hydrogels (5, 10, and 20 wt%) and 10 wt% PEG hydrogels as controls. Four replicates for each testing condition were prepared for both characterization methods. For stress relaxation, the shear stress was monitored over 10 min at a constant strain (15, 45, and 90%) for each RLP and PEG hydrogel, and the data was fitted to an exponential decay function to calculate the relaxation rate. For creep experiments, shear strain was monitored the first 20 min after a shear stress (ranging from 50 to 4000 Pa) was applied, and followed by another 20 min when the shear stress was removed, to explore the percentage of strain recovered upon removal of applied stress.

### Uniaxial tensile testing

RLP films for uniaxial mechanical testing were prepared in contact lens molds (Bausch and Lomb, Rochester, NY) by the addition of desired amounts of THP to 20 wt% RLP in PBS buffer. The hydrogel films were cross-linked at 37°C for 2 h. Before the measurements, hydrogel films were hydrated in PBS overnight to reach equilibrium and cut into dog-bone specimens with a stainless steel mold (width 2 mm; length 6 mm). (The PEG hydrogels could not be characterized via tensile testing owing to the poor mechanical properties of the PEG hydrogels.) The test samples were mounted on an Instron 4502 mechanical tester equipped with a 250 g mechanical load cell and were tested at room temperature under hydrated conditions, utilizing a tank containing saline buffer around the grips. For stress relaxation experiments, three replicates of RLP hydrogel samples were stretched to 15, 30, 60, and 100% strain at a constant strain rate of 5 mm/min, and then the stress was monitored over 10 min at each fixed strain. For cyclic loading and unloading experiments, stress-strain data were recorded at the same strain rate and the hydrogel films were subjected to three cycles each to strains of 30, 60, and 100%, and then to failure. Resilience values were obtained by dividing the area under the unloading curves by the area under the loading curves at each fixed cycle; average values were obtained from at least three replicate samples. Young's modulus values were calculated from the linear region (5–15%) of the stress-strain curve.

## Results and discussion

### Expression and purification of RLPs

**Figure 1:**
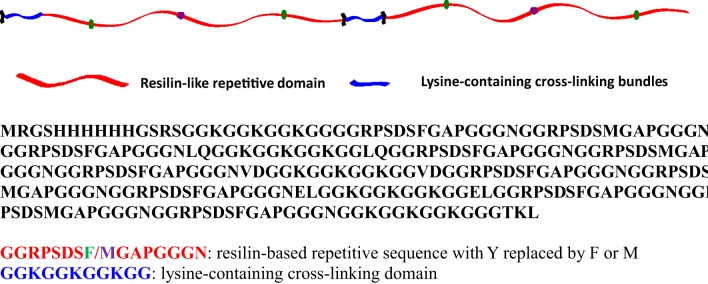
**Schematic of two repeats of the RLP, and the entire amino acid sequence of the RLP employed in these studies**.

The detailed amino acid sequence of the RLP is presented in Figure 1. Twelve repeats of the pro-resilin putative consensus sequence (GGRPSDSFGAPGGGN, derived from the first exon of Drosophila melanogaster CG15920) was employed (Charati et al., 2009; Li et al., 2011, 2013; McGann et al., 2013). Five lysine-containing GGKGGKGGKGG cross-linking bundles were incorporated and placed evenly along the polypeptide chain, with 45 amino acids in between, to mimic the cross-link density in natural resilin (ca. 30–60 amino acids between di- and tri-tyrosine crosslinks) (Andersen, 1964; Elliott et al., 1965). The expression of RLP, via traditional IPTG induction, yielded 20–30 mg/L protein per liter of cell culture. Cell pellets were lysed under native conditions, followed by washing steps and elution from a Ni-NTA column in native buffer, followed by dialysis against DI H_2_O to remove salts before lyophilization. The molecular weight (23 kDa), purity (>95%), and final composition of the RLP samples were confirmed via SDS-PAGE, MALDI-TOF MS, HPLC, and amino acid analysis (Li et al., 2013).

### Oscillatory rheological characterization of RLP and PG hydrogels

*In situ* oscillatory shear rheology was employed to monitor the viscoelastic properties of the RLP hydrogels and provided a practical approach to characterize the impact of crosslinking conditions on gelation kinetics, gel stability and ultimate equilibrium shear moduli of hydrogels. Guided by the vocal fold ECM composition and distribution *in vivo*, we chose RLP hydrogels at 5, 10, and 20 wt% material concentrations, and characterized the RLP materials under physiologically relevant strain (15–90%) and stress (50–4000 Pa) ranges (Jiang et al., 2000; Kutty and Webb, 2009b). The hydrogels were cross-linked under mild aqueous conditions via the Mannich-type condensation reaction of THP with primary amines from the lysine residues of polypeptide. Protein and polymer solutions were pre-vortexed with THP solutions, to ensure homogeneous mixing, at room temperature before deposition of the solution onto the Peltier plate to form hydrogels, at an equimolar ratio of reactive lysines to HMP groups.

**Figure 2:**
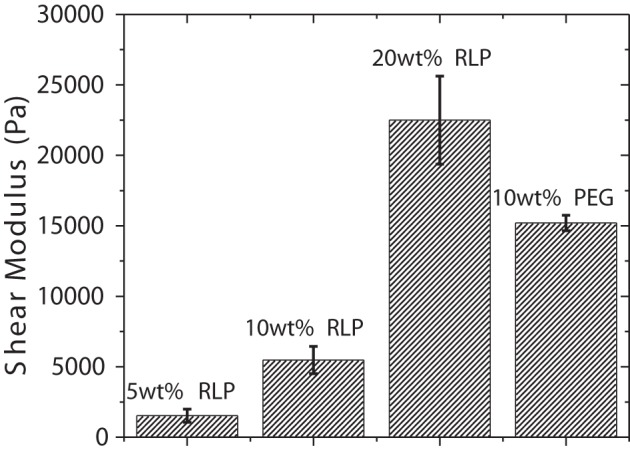
**Shear storage moduli of RLP and PEG hydrogels**. Oscillatory time sweeps for *in situ* cross-linking of RLP and PEG hydrogels were conducted for 2 h at 37°C at a frequency of 6 rad/s and at1% strain. The final equilibrium storage modulus (G′) for each composition is reported. Error bars represent the standard deviation of three measurements.

**Figure 3:**
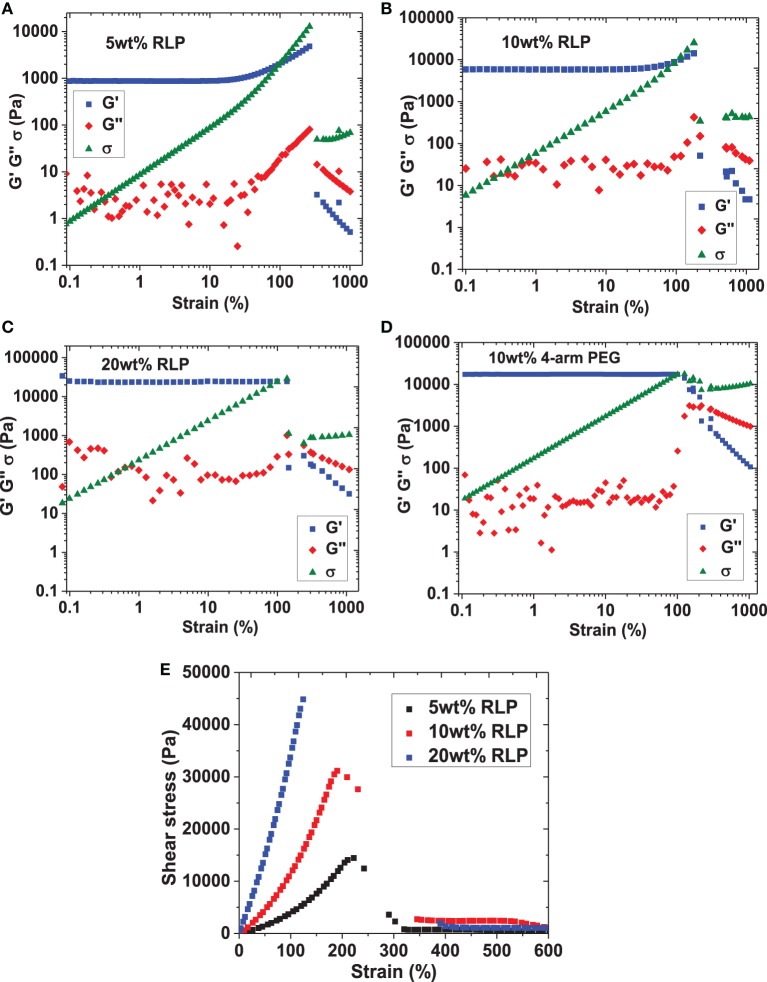
**Strain sweeps of RLP hydrogels at (A) 5wt%, (B) 10wt%, and (C) 20wt% polypeptide concentrations and (D) 10wt% 4-arm PEG hydrogels. (E)** Summary of steady shear flow, strain-to-break experiments on all RLP compositions, yielding similar strain-to-break values compared to those collected under oscillatory mode (5 wt%: 220 ± 7%; 10 wt%: 200 ± 6%; 20 wt%: 150 ± 20%).

The shear mechanical moduli of cross-linked RLPs were characterized *in situ* via dynamic oscillatory shear mode rheometry using a cone-on-plate geometry after cross-linking the hydrogels under physiologically relevant conditions. Samples tested using two cone-on-plate geometries with different cone-angles resulted in similar gelation times, times to plateau, and final storage moduli, confirming the lack of slip during these dynamic oscillatory shear rheological experiments. Both RLP- and PEG-hydrogels were characterized in time sweep, frequency sweep, and strain sweep modes; the equilibrium shear storage moduli (G′) and strain sweep profiles for the various hydrogels are presented in Figure S1, Figures 2, 3. During the time sweep experiment, all polypeptide solutions exhibited fast gelation, at 37°C, upon mixing with THP cross-linker at 1:1 stoichiometric ratio; shear storage moduli reached an equilibrium value within 10 min with 50–100-fold differences (data not shown) between the storage modulus (G′) and loss modulus (G″), indicating the formation of solid elastic hydrogels. Increasing protein concentration increased the rate of gelation and also resulted in increased final storage modulus values, as expected; RLP hydrogels of concentrations at 5, 10, and 20 wt% yielded storage modulus values of 1, 5, and 20 kPa, while a 10 wt% PEG control hydrogels yielded a storage modulus of 15 kPa (Figure 2). The 10 wt% PEG hydrogel was chosen not only to match the polymer concentration of the 10 wt% RLP hydrogel, but also to achieve a G′ value between those of the 10 and 20 wt% RLP hydrogels.

Dynamic oscillatory frequency sweeps ranging from 0.1 to 100 rad/s were then employed to assess the stability of RLP- and PEG-hydrogels; the insensitivity of shear moduli over the frequency range investigated confirmed the hydrogels behave as elastic solid-like materials derived from permanently cross-linked networks (data not shown). Finally, oscillatory shear strain sweeps from 0.1 to 1000% at 6 rad/s were characterized to illustrate the yield behavior of RLP- and PEG-hydrogels over various compositions; the results of these experiments are summarized in Figure 3. As shown in the figure, the linear regime extends to approximately 30, 60, 100, and 100% strain for 5, 10, 20 wt% RLP hydrogels and the 10 wt% 4-arm PEG hydrogel, respectively (Figures 3A–D). For the RLP-based hydrogels, further increases in strain amplitude result in an increase in both the storage (G′) and loss moduli (G″) until they cross-over (G″ > G′), with a sharp overall decrease in the stress response resulting from failure of the gel network. The strain stiffening observed in these hydrogels is consistent with that observed in multiple other biological hydrogels (including native vocal folds) as well as in other synthetic networks (Xu and Craig, 2011; Xu et al., 2012); strain stiffening in biological tissues may serve as a means to prevent tissue damage from exposure to large deformation (Storm et al., 2005; Erk et al., 2010). Quantitatively, the average oscillatory shear strain-to-break values are 240 ± 30%, 190 ± 20%, and 160 ± 20% for the 5, 10, and 20 wt% RLP hydrogels, respectively. The PEG hydrogels did not exhibit a sharp decrease in the amplitude of the shear stress [even though G″ exceeds G′ (Figure 3D)], suggesting slipping of materials under large strain. All subsequent stress relaxation and creep experiments were conducted well below 100% strain, where no significant slip was indicated. The hydrogels were also tested under continuous shear in steady shear flow, strain-to-break experiments; the data from these experiments are shown in Figure 3E. Although these conditions do not mimic the mechanical environment of human vocal folds *in vivo*, the data provide additional information to compare strain-to-break properties of RLP hydrogels to the data acquired from oscillatory experiments. As shown in the figure, the strain-to-break values for RLPs are 220 ± 7%, 200 ± 6%, and 150 ± 20% at 5, 10, and 20 wt% polypeptide concentrations, which are in good agreement with results from dynamic shear strain sweeps.

### Oscillatory shear stress relaxation

The time-dependent mechanical properties of tissues are important to their function and repair. For example, the repair of vocal fold tissue should benefit from the match of stress-relaxation behavior to that of native tissue, especially during phonation, to permit stimulation of appropriate cellular behavior with desired matrix deposition. In matrices intended for drug delivery, the application of controlled mechanical strain over select periods of time can be useful for regulating the release of drugs (Titze, 1988; Chan and Titze, 1999, 2000; Zhang et al., 2009; Xiao et al., 2013). In order to further explore the energy dissipation behavior of RLP-based hydrogels, the oscillatory shear stress relaxation of *in situ* cross-linked RLP hydrogels was characterized; the relaxation behavior of these materials is relevant owing to the importance of transient mechanical responses of the vocal fold tissue in phonation.

**Figure 4:**
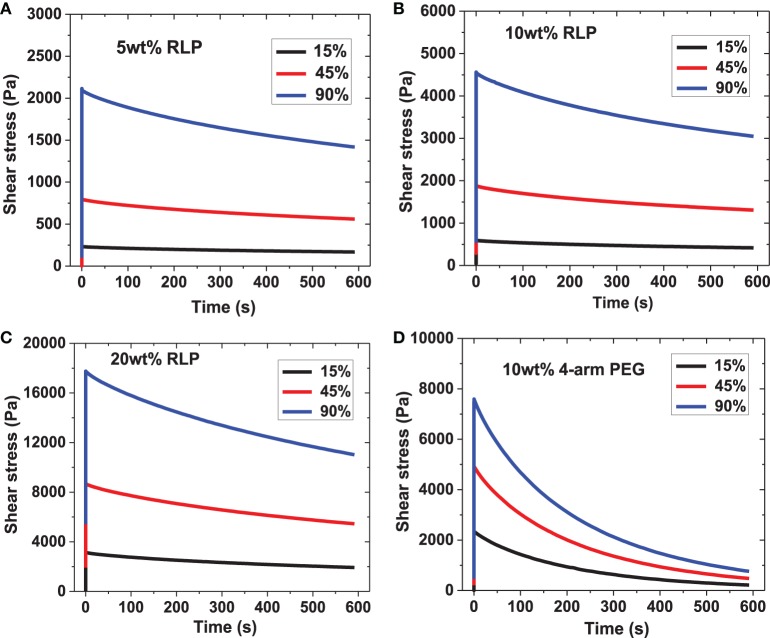
**Representative oscillatory stress relaxation data. (A)** 5 wt% RLP, **(B)** 10 wt% RLP, **(C)** 20 wt% RLP and **(D)** 10 wt% 4-arm PEG cross-linked hydrogels at 15, 45, and 90% strain.

Shear stress relaxation experiments were conducted on 5, 10, and 20 wt% RLP hydrogels and the 10 wt% PEG control hydrogel. After hydrogel formation was complete (indicated by G′ reaching a plateau), each sample was subjected, consecutively, to 15%, then 45%, and finally 90% strain values, for 10 min at each strain. The oscillatory shear stress was monitored over time; representative data are shown in Figure 4. When the cross-linked RLP hydrogels were deformed to 15, 45, and 90% strain, the shear stress quickly increased and reached a maximum value; this behavior was observed across all polypeptide concentrations and applied strains (Figures 4A–C). This essentially instantaneous increase of shear stress demonstrates the highly elastic response of RLP-hydrogels to external energy input, as anticipated for an ideal rubber. The shear stress was then monitored as a function of time for 10 min under constant strain; this time period was chosen based upon the fact that the time scale is long enough to observe any stress relaxation from dynamic protein chain movements as well as to maintain experimental conditions similar to those reported for other recombinant or natural proteins. The 5, 10, and 20 wt% RLP hydrogels exhibited similar stress relaxation behavior (Figures 4A–C), with a largely elastic response with relatively minor stress relaxation after each incremental strain. This behavior is similar to the stress relaxation response reported for canine vocalis muscle tissue (Alipour-Haghighi and Titze, 1985). The stress relaxation of the 10 wt% PEG hydrogel (Figure 4D), however, was markedly different. Although the PEG hydrogels also exhibited an immediate increase in stress similar to that observed for the RLP hydrogels (as expected for the chemically cross-linked network), the PEG hydrogel network was not able to hold the energy input over time and displayed a significant decrease in shear stress under the same experimental conditions. These differences are suggested to arise from the intrinsic disparities in the properties of the polymer chains of these two systems, given that the crosslinking densities and chemistries are similar between the PEG control and the RLP hydrogels. Among the RLP hydrogels, a slight increase in stress relaxation is observed with an increase in RLP concentration, suggesting that the higher-concentration samples are less elastomeric, consistent with the reported importance of hydration in the elastomeric behavior of RLPs (Gosline et al., 2002; Truong et al., 2011).

**Figure 5:**
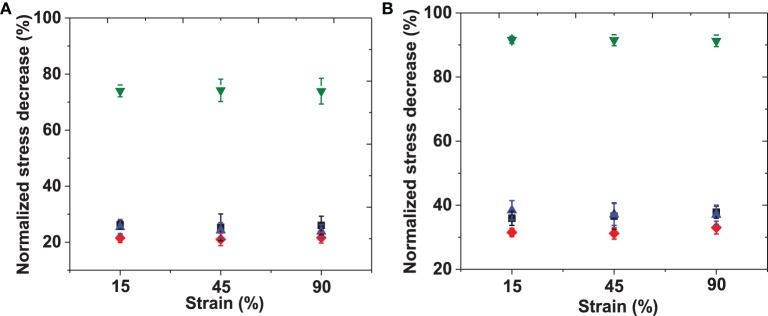
**Percentage of normalized shear stress decreased at (A) 100 s and (B) 600 s**. Shear stresses at each strain were normalized to the shear stress at *t* = 0 min of 5 wt% (black), 10 wt% (red), 20 wt% (blue) RLPs and 10 wt% 4-arm PEG hydrogels (green) at 15, 45, and 90% constant strains. Error bars represent the standard deviation of three measurements.

In order to facilitate comparison of the data, the shear stresses for all materials recorded at time 0, 100, and 600 s were tabulated. The stresses at later time-points were compared to the initial stress and the percentage of shear stress lost was calculated; the averaged data are listed in Table S1 and illustrated graphically in Figure 5. The short-scale (100 s) and long-time (600 s) intervals were chosen to facilitate the comparison to reported stress relaxation properties of other previously reported materials. The initial shear stress (*t* = 0 s) upon input of elastic energy increased with an increase in applied strain, and with RLP hydrogel concentration, as expected. The RLP hydrogels exhibited a relatively minor reduction in stress compared to the initial shear stress, with reductions of approximately 10% at 100 s (Figure 5A) and 35% at 600 s (Figure 5B), over all three RLP hydrogel concentrations and all applied strains. In contrast, the PEG hydrogels exhibited almost 35 and 90% reduction in shear stress at 100 and 600 s. Although select other PEG-based materials have shown less stress relaxation than observed here, the reduced stress relaxation was observed in systems of higher polymer concentrations, different cross-linking chemistries, and tightly cross-linked networks mixed with multiple components (Snyders et al., 2007; Roberts et al., 2011; Cui et al., 2012).

**Figure 6:**
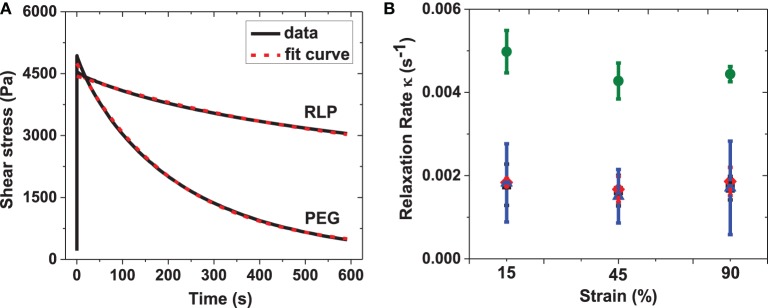
**(A)** Representative fitting curves for 10 wt% RLP and 10 wt% 4-arm PEG hydrogels; **(B)** summary of relaxation rate of 5 wt% (square), 10 wt% (diamond) and 20 wt% (triangle) RLP and 10 wt% 4-arm PEG (circle) hydrogels at 15, 45, and 90% constant strains. The stress relaxation behavior was fitted to an exponential equation: σ (t) = σ_o_ + A^*^ exp(− *t*κ), where σ_o_ is the offset shear stress, A is the decay amplitude and κ is the relaxation rate. Error bars represent the standard deviation of three measurements.

In order to quantitatively analyze the stress relaxation, the stress relaxation curves of RLP and PEG hydrogels were fit to a single exponential equation: σ (t) = σ_o_+ A^*^ exp( − tκ), where σ_o_ is the offset shear stress, A is the decay amplitude, and κ is the relaxation rate. Representative stress relaxation curves and fits for the 10 wt% RLP and 10 wt% PEG hydrogels are shown in Figure 6A, illustrating the good fit of the data to a single exponential decay. The relaxation rate constants for all hydrogels are compared in Figure 6B. For RLP hydrogels, the relaxation rates fall within the range of 0.001–0.002 s^−1^ across all hydrogel concentrations and applied strains, while the relaxation rates for the PEG hydrogel are twice as fast (approximately 0.0045 s^−1^). The relaxation rates for the RLP-based hydrogels reported here are 10-fold slower than those reported for GB1-RLP multi-domain proteins, which feature a folded streptococcal B1 immunoglobulin-binding domain of protein G (GB1 domain) alternating with random-coil resilin domains, in order to mimic the muscle protein titin (Cao and Li, 2007; Lv et al., 2010). This difference is likely due to the fact that the RLP hydrogels lack folded protein domains; in the GB1-RLP materials, single RLP consensus motifs are intercepted by multiple GB1 folded domains. The rapid response of the RLP hydrogels with an immediate increase in shear stress, followed by a minor loss in shear stress, demonstrates the efficient response to energy input of the RLP materials, with low energy dissipation. This behavior is similar to that observed in ideal rubbers and vocal fold tissues (Alipour-Haghighi and Titze, 1985; Urry et al., 2002), and substantially different than that observed for PEG-control hydrogels and other reported polysaccharide-based and ELP-based hydrogels (Cloyd et al., 2007; Shazly et al., 2008; Wu et al., 2008; Riede et al., 2011; Roberts et al., 2011; Krishna et al., 2012; Razavi-Nouri, 2012).

### Oscillatory creep measurements

**Figure 7:**
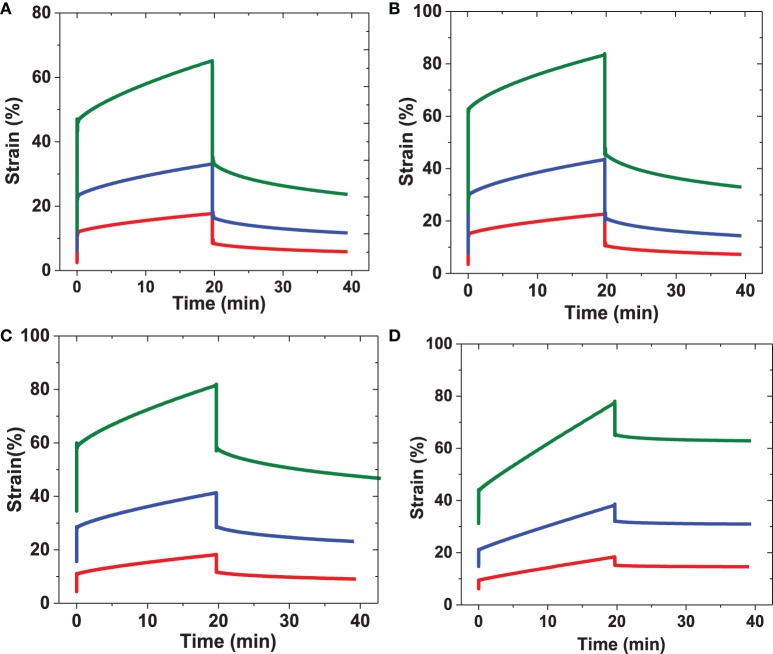
**Representative creep behavior for RLP and PEG hydrogels**. Samples were subjected to applied constant shear stresses for 20 min followed by removing the applied shear stresses and monitoring strain change over another 20 min. **(A)** 5 wt% RLP at constant shear stress 100 Pa(red), 200 Pa(blue), and 500 Pa(green); **(B)** 10 wt% RLP at constant shear stress 500 Pa(red), 1000 Pa(blue), and 2000 Pa(green); **(C)** 20 wt% RLP at constant shear stress 1000 Pa(red), 2000 Pa(blue), and 4000 Pa(green) and **(D)** 10 wt% 4-arm PEG cross-linked hydrogels at constant shear stress 500 Pa(red), 1000 Pa(blue), and 2000 Pa(green).

Time-dependent changes in strain response to stress are influenced by the density and pore size of a 3D network, its hydration, as well as the chemical nature of the cross-links. For example, it has been reported that ELP-based biomaterials show substantially different deformation responses depending on the conditions under which they are tested. Variations in temperature, solvent, and preconditioning steps have been shown to influence their deformation. Specifically, ELP-films cast below the transition temperature or in 2,2,2-trifluoroethanol (TFE) solvent displayed limited creep response (<10%) compared to films cast above the transition temperature in water (creep>60%) (Wu et al., 2008). Creep analysis under oscillatory rheology conditions was conducted on RLP and PEG hydrogels to explore the transient dynamic mechanical responses; both the instant and total strain deformation (at 20 min) and strain recovery (after 20 min, σ = 0 Pa) under various constant shear stresses were characterized. Representative data from the hydrogels are shown in Figure 7. We note that although the strain deformation was kept similar across the various samples to permit comparisons of strain recovery, the applied stresses were different for the various RLP and PEG hydrogels, as higher stresses were necessary to achieve the same strain deformation on hydrogels with a higher shear modulus. For example, approximately 45% instant strain was observed for a 5 wt% RLP hydrogel upon application of a shear stress of 500 Pa; however, application of a shear stress of 2000 Pa was required in order to obtain percentage similar strain deformation for the 10 wt% PEG hydrogel.

**Figure 8:**
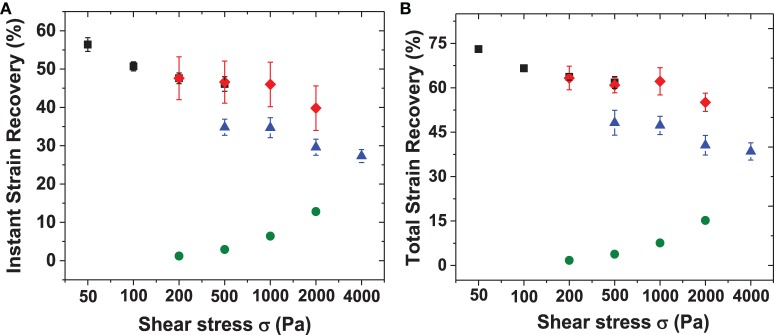
**Summary of instant and total strain recovery for RLP and PEG hydrogels upon removing the applied shear stresses**. Both instant and total strain recoveries were normalized to the highest strain reached before removal of the applied shear stresses. **(A)** Instant stain recovery at 20 min and **(B)** total strain recovery at 40 min for 5 wt% (square), 10 wt% (diamond), 20 wt% (triangle) RLP-based hydrogels and 10 wt% 4-arm PEG-hydrogels (circle) with applied constant shear stresses ranging from 50 to 4000 Pa.

The creep data for RLP hydrogels at 5, 10, and 20 wt% concentrations are shown in Figures 7A–C, with summaries of the data given in Figure 8, and Table S2. The various hydrogels exhibit similar creep behaviors, with large instantaneous deformation followed by a slow increase in strain over 20 min with constant applied shear stress. Significant strain recovery was observed for all hydrogels when the applied shear stress was removed; the hydrogels continued to recover over the next 20 min with total strain recovery of approximately 70, 60, and 50% for the 5, 10, and 20 wt% RLP hydrogels, respectively. In comparison, the 10 wt% PEG-based hydrogel control (Figure 7D) displayed a lower instant strain deformation followed by a sharper increase of strain over 20 min at constant applied shear stress. Moreover, minimal strain recovery was observed upon the removal of the applied shear stress, with only small strain recovery (15%) over the following 20 min. Generally speaking, the RLP and PEG hydrogels exhibited creep behavior typical of viscoelastic materials, although show distinct differences in immediate and total strain recovery (Figure 8 and Table S2). The highly dynamic behavior of the flexible RLP chains is reported to be derived from hydrogen bond exchange, charge interactions, and the high polarity of the side chains; the elastomeric properties of the RLP-based materials here is consistent with this behavior.(Kappiyoor et al., 2011) The applied shear stresses (50–4000 Pa) in our experiments, selected for deformation of the RLP-based hydrogels to physiologically relevant strains, are significantly lower than those applied (30 KPa–0.8 MPa) in the characterization of elastin-like polypeptide- (ELP) and silk-elastin like polypeptide(SELP)-based biomaterials (McPherson et al., 1992; Wu et al., 2008; Qiu et al., 2009, 2010; Teng et al., 2009), owing to the fact that the RLP-based hydrogels are more soft and extensible than the previously reported materials.

### Uniaxial tensile testing

**Figure 9:**
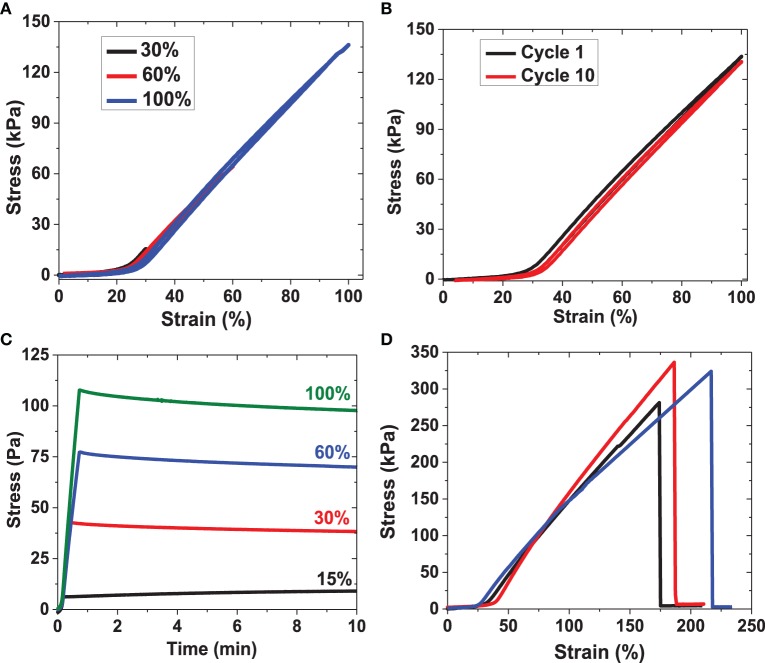
**Uniaxial tensile testing experiments for RLP hydrogels. (A)** Third cycle of strain-loading and unloading up to 30, 60, and 100% strain for RLP hydrogel; **(B)** first cycle and tenth cycle of cyclic loading and unloading at 100% strain of RLP hydrogel; **(C)** stress relaxation of RLP hydrogel at 15, 30, 60, and 100% constant strains; **(D)** three repeats of strain-to-break tensile testing experiment on RLP hydrogels. All materials characterized in tensile testing experiments are 20 wt% hydrated RLP hydrogels at 1:1 cross-linking ratio.

Repeated loading and unloading strain cycles in standard tensile testing format were employed to probe the deformation response, analyze hysteresis, determine the longitudinal elasticity, and calculate the resilience of hydrated RLP-based hydrogels comprising polypeptide sequences and numbers of cycles that are distinct from those of our previous reports. Twenty wt% RLP hydrogel films were the only samples employed in the uniaxial tensile testing experiments due to practical limitations in the handling of the softer RLP and PEG hydrogels; representative data from these experiments are shown in Figure 9.

For each sample, three consecutive loading and unloading cycles were applied at each strain, first to 30% (three cycles), then to 60% (three cycles), and then to 100% strain (three cycles); only the third cycles at each strain are shown in Figure 9A for simplicity. Resilience values were calculated by dividing the area under the unloading curves by the area under the loading curves at each cycle. The overlap of the stress-strain curves during loading and unloading confirms the negligible hysteresis of these RLP hydrogels, indicating their reversible recovery and excellent elasticity even up to 100% strain over many types of compositions (Elvin et al., 2005; Charati et al., 2009; Lyons et al., 2009; Li et al., 2011; Qin et al., 2011). A non-linear dependence of stress with applied strain is observed, similar to previously reported data for both human vocal ligament and the vocal fold cover (Min et al., 1995; Zhang et al., 2006; Chan et al., 2007). Ten consecutive loading and unloading cycles were conducted to 100% strain; only the first and the 10th cycle are plotted for comparison in Figure 9B. The two stress-strain curves overlap almost perfectly with resilience values approaching 95%, indicating the excellent recovery and fatigue resistance of the RLP hydrogel films, confirming the elastic storage and release of the energy introduced during the repeated extension process, and demonstrating that negligible energy was dissipated as heat during the deformation.

For the stress relaxation experiment, RLP hydrogels were stretched to 15, 30, 60 and 100% strain at a 5 mm/min strain rate. The length of the sample remained constant while the stress was monitored as a function of time; representative data are shown in Figure 9C. The stress increased rapidly once the strain was applied to the hydrogels, indicating instantaneous deformation, as in the oscillatory experiments. Essentially negligible stress-relaxation, consistent with the behavior of an ideal rubber, was observed for 20 wt% RLP hydrogels at the various strains tested, in sharp contrast to the significant stress relaxation observed for previously reported ELP-based hydrogels and GB1-RLP proteins, (Wu et al., 2008; Teng et al., 2009; Lv et al., 2010). The significant stress relaxation in the latter materials results from irreversible unfolding of the protein chains with applied stress, suggesting the absence of such folding/unfolding in the cross-linked RLP hydrogels (Lv et al., 2010).

Strain-to-break experiments on these RLP hydrogels were also conducted to determine the longitudinal elasticity and Young's modulus. As shown in Figure 9D, hydrated RLP films show an average extension-to-break value of approximately 190% with an average Young's modulus value of approximately 30 kPa. The RLP-based hydrogels exhibited linear stress-strain behavior at low strain (0-15%) and non-linear behavior at high strain, similar to the tensile behavior observed for human vocal ligaments, (Min et al., 1995; Kutty and Webb, 2009a).

A detailed summary of the results from the tensile testing, including resilience values, Young's modulus, and average strain-to-break values, is provided in Table S3. The average strain-to-break values (190 ± 20%) for the RLP hydrogel (cross-linking efficiency of ca. 40% and water content of ca. 80%), are consistent with the properties reported for natural resilin and other recombinantly synthesized RLPs characterized under similar strain rates (Weis-Fogh, 1960; Elvin et al., 2005; Wu et al., 2008; Charati et al., 2009; Lyons et al., 2009; Lv et al., 2010; Li et al., 2011, 2013; Qin et al., 2011; Renner et al., 2012; McGann et al., 2013). The Young's modulus of the RLP-based hydrogel films is approximately 30 kPa, which compares favorably to those of human vocal fold tissues (20–40 kPa) and other RLPs (25.5 kPa). The RLP-based hydrogels displayed high resilience values ranging exceeding 95% over repeated strain cycles, which also is consistent with natural resilin and previously reported resilin-based polypeptides, and improved over available data reported for ELP-based hydrogels (Daamen et al., 2007; Wu et al., 2008; Krishna et al., 2012). Slight hysteresis between the first and subsequent cycles is often observed, and is likely due to stabilization of load-induced changes in microstructure, such as the dissociation of entanglements, after initial stretching (Nagapudi et al., 2005; Wu et al., 2008). The heterogeneity in the strain-to-break values might arise from sample specific local defects, micro-cracks, sample gripping, loading, and variations of sample thickness. The hydrated RLP hydrogels exhibited improved elastomeric properties over those for the hydrogels formed *in situ* in the oscillatory rheology experiments; this is probably due to the fact that water behaves as a lubricant for the RLP polymer networks by enhancing the exchange of hydrogen bonds and reducing charge interactions between polar amino acid side chains thus increasing chain flexibility. These dynamic features of the polypeptide chain promote conformational changes that further dissipate the energy input, lowering the stiffness and providing a higher resistance to fracture (Gosline et al., 2002; Nairn et al., 2008; Woody, 2009; Kappiyoor et al., 2011; Truong et al., 2011). Taken together, the outstanding elastomeric mechanical properties of the RLP hydrogels, under various loading conditions, recommend the potential use of RLP-based hydrogels for vocal fold tissue regeneration applications.

## Conclusions

Given the excellent mechanical properties of naturally occurring resilin, recombinant RLPs have been designed and studied for vocal fold tissue engineering applications. This modular RLP allows efficient gelation upon mixing with the cross-linker THP, and exhibits mechanical stability and extensibility. Facile tuning of mechanical properties (e.g., storage modulus) can be achieved by changing the RLP concentrations of hydrogels. *In situ* oscillatory shear stress relaxation experiments illustrated that RLP-based hydrogels showed substantially improved energy storage over that observed for PEG-based hydrogels. Similarly, oscillatory creep experiments demonstrated that RLP-based hydrogels exhibited larger deformation and subsequently more strain recovery compared to those of PEG-based hydrogels. Uniaxial tensile testing showed negligible hysteresis, reversible elasticity, and superior resilience (up to 98%) of hydrated RLP hydrogels, with Young's modulus values comparing favorably with those previously reported for resilin and mimicking the tensile properties of the vocal fold ligament at low strain. Together, the data presented here highlight the relevance of the dynamic mechanical properties of these RLP-based materials, which are highly comparable to those of targeted vocal fold tissue, and illustrate the opportunities for creating scaffolds of unique applicability to vocal fold tissue engineering.

### Conflict of interest statement

The authors declare that the research was conducted in the absence of any commercial or financial relationships that could be construed as a potential conflict of interest.

## Supplementary material

Figure S1 **Dynamic oscillatory shear storage moduli of RLP hydrogels at 5, 10, and 20 wt% concentrations**. Oscillatory time sweeps were conducted for 2 h at 37°C at a frequency of 6 rad/s and at 1% strain.

Table S1 **Summary of average shear stress and normalized decrease in shear stress at ***t*** = **0**, 100, 600 s with constant strain at ε = **15**, 45, and 90% amplitude for 5, 10, 20 wt% RLP and 10 wt% 4-arm PEG hydrogel compositions**. Errors reported are the standard deviation from a minimum of four measurements.

Table S2 **Summary of average strain deformation and normalized strain recovery upon release of applied shear stress.** Average strains (@ *t* = 0 min and 20 min) upon deformation with applied shear stress ranging from 50 to 4000 Pa were calculated from the data of multiple samples. Normalized percentages of strain recovery after 20 min (immediate recovery) and 40 min (total recovery) were calculated for 5, 10, 20 wt% RLP and 10 wt% 4-arm PEG hydrogel compositions. Errors are reported as the standard deviation from a minimum of four measurements.

Table S3 **Summary of general properties and resilience values for 20 wt% RLP hydrogels**.
