# Supplementary material for: Transient dynamic mechanical properties of resilin-based elastomeric hydrogels
Source: Front Chem. 2014 Apr 28;2:21. doi: 10.3389/fchem.2014.00021 (PMC4009447; doi:10.3389/fchem.2014.00021)
Supplement: Figure S1 — Dynamic oscillatory shear storage moduli of RLP hydrogels at 5, 10, and 20 wt% concentrations. Oscillatory time sweeps were conducted for 2 h at 37°C at a frequency of 6 rad/s and at 1% strain. [file DataSheet1.DOCX]

***Supplementary Material***

**Transient Dynamic Mechanical Analysis of Resilin-based Elastomeric Hydrogels**

**Linqing Li^1^, Kristi L. Kiick^1,2,3^***

^1^Department of Materials Science and Engineering, University of Delaware, Newark, Delaware, 19716, United States

^2^Biomedical Engineering, University of Delaware, Newark, Delaware, 19716, United States

^3^Delaware Biotechnology Institute, 15 Innovation Way, Newark, Delaware, 19711, United States

*** Correspondence:** Kristi L. Kiick, Department of Materials Science and Engineering, University of Delaware, Newark, Delaware, 19716, United States.

kiick@udel.edu

**Figure S1. Dynamic oscillatory shear storage moduli of RLP hydrogels at 5wt%, 10wt% and 20wt% concentrations.** Oscillatory time sweeps were conducted for two hours at 37 ^o^C at a frequency of 6 rad/s and at 1% strain.

**Table S1. Summary of average shear stress and normalized decrease in shear stress at t=0, 100, 600 seconds with constant strain at ε=15%, 45% and 90% amplitude for 5wt%, 10wt%, 20wt% RLP and 10wt% 4-arm PEG hydrogel compositions.**

| Strain (%) | Shear stress  (Pa) | | | Percentage Decreased  (%) | |
| --- | --- | --- | --- | --- | --- |
|  | **t=0s** | **t=100s** | **t=600s** | **@100s** | **@600s** |
| 5wt% RLP | | | | | |
| 15% | **260±20** | **220±10** | **160±10** | **14±2** | **36±2** |
| 45% | **900±90** | **780±20** | **560±20** | **14±5** | **37±4** |
| 90% | **2270±150** | **1970±30** | **1390±40** | **14±3** | **38±2** |
| 10wt% RLP | | | | | |
| 15% | **600±20** | **540±20** | **430±20** | **9±2** | **32±1** |
| 45% | **1940±170** | **1760±130** | **1360±120** | **9±2** | **31±2** |
| 90% | **4580±250** | **4130±170** | **3070±130** | **10±2** | **33±2** |
| 20wt% RLP | | | | | |
| 15% | **2930±290** | **2590±190** | **1840±140** | **12±3** | **38±3** |
| 45% | **8160±410** | **7284±500** | **5190±380** | **11±2** | **37±3** |
| 90% | **17000±220** | **15170±410** | **10680±480** | **11±3** | **37±3** |
| 10wt% 4-arm PEG | | | | | |
| 15% | **2340±30** | **1430±40** | **200±30** | **39±2** | **92±1** |
| 45% | **4850±110** | **3040±170** | **410±90** | **37±4** | **92±2** |
| 90% | **7400±210** | **4700±280** | **650±160** | **37±5** | **91±2** |

Errors reported are the standard deviation from a minimum of four measurements.

**Table S2. Summary of average strain deformation and normalized strain recovery upon release of applied shear stress.**

| **Shear Stress** | **Strain Deformation (%)** | | | **Strain Recovery (%)** | |
| --- | --- | --- | --- | --- | --- |
| **σ** | | **t=0 min** | **t=20 min** | **t=20min, σ=0** | **t=40min, σ=0** |

| 5wt% RLP | | | | |
| --- | --- | --- | --- | --- |
| 50Pa | **5±1** | **9±1** | **56±2** | **73±0** |
| 100Pa | **12±1** | **18±2** | **51±1** | **67±1** |
| 200Pa | **23±2** | **33±3** | **48±1** | **64±1** |
| 500Pa | **46±5** | **65±5** | **46±2** | **62±2** |
| 10wt% RLP | | | | |
| 200Pa | **5±1** | **8±1** | **48±6** | **63±4** |
| 500Pa | **13±2** | **20±2** | **47±7** | **61±3** |
| 1000Pa | **25±3** | **38±4** | **46±6** | **62±5** |
| 2000Pa | **56±6** | **77±7** | **40±5** | **55±5** |
| 20wt% RLP | | | | |
| 500Pa | **6±1** | **9±0** | **35±2** | **48±4** |
| 1000Pa | **12±1** | **19±1** | **35±3** | **47±4** |
| 2000Pa | **30±1** | **43±1** | **30±2** | **41±3** |
| 4000Pa | **61±3** | **85±3** | **27±2** | **39±3** |
| 10wt% 4-arm PEG | | | | |
| 200Pa | **5±1** | **9±1** | **1±0** | **2±0** |
| 500Pa | **12±3** | **21±2** | **3±0** | **4±0** |
| 1000Pa | **22±2** | **41±3** | **6±0** | **8±0** |
| 2000Pa | **47±3** | **82±6** | **13±1** | **15±1** |

Average strains (@ t=0 min and 20 mins) upon deformation with applied shear stress ranging from 50 to 4000Pa were calculated from the data of multiple samples. Normalized percentages of strain recovery after 20 mins (immediate recovery) and 40 mins (total recovery) were calculated for 5wt%, 10wt%, 20wt% RLP and 10wt% 4-arm PEG hydrogel compositions. Errors are reported as the standard deviation from a minimum of four measurements.

**Table S3. Summary of general properties and resilience values for 20wt% RLP hydrogels**.

| **Composition** | **Swelling ratio**  **(Q)** | | **Water content**  **(%)** | **Cross-linking efficiency (%)** | **Young’s modulus (kPa)** | | **Strain-to-break (%)** |
| --- | --- | --- | --- | --- | --- | --- | --- |
| **RLP** | **5.2±0.1** | | **80.6±0.4** | **42.6±0.9** | **30.3±8.1** | | **190±20** |
| **Cycles/Strain** | | **30%** | | **60%** | | **100%** | |
| **Cycle-1** | | **94.0±0.7** | | **94.0±0.9** | | **94.4±0.6** | |
| **Cycle-2** | | **98.4±0.9** | | **95.8±0.6** | | **95.4±0.3** | |
| **Cycle-3** | | **98.8±1.2** | | **96.3±0.6** | | **95.6±0.3** | |
